# Related Risk Factors of Early Childhood Caries in Zhejiang Province, China During the COVID-19 Pandemic

**DOI:** 10.3389/fpubh.2022.879955

**Published:** 2022-09-30

**Authors:** Chaoqiang Huang, Kangqi Zhu, Yibing Feng, Luya Lian, Haihua Zhu, Jian Hu

**Affiliations:** ^1^The Stomatological Hospital of Zhejiang Chinese Medicine University, Hangzhou, China; ^2^Key Laboratory of Oral Biomedical Research of Zhejiang Province, Clinical Research Center for Oral Disease of Zhejiang Province, Cancer Center of Zhejiang University, Stomatology Hospital, School of Stomatology, Zhejiang University School of Medicine, Hangzhou, China

**Keywords:** early childhood caries, district economic level, desserts consumption, risk factors, oral epidemiological investigation

## Abstract

**Objectives:**

This work aims to examining the latest early childhood caries situation in children aged 3 and 5 and its related risk factors in Zhejiang Province during the COVID-19 pandemic.

**Method:**

There are 3,537 children and their main caregivers participate in this study. We used chi square test or U-test to analyze whether there were differences in the prevalence of dental caries under different variables. The risk factors on ECC were determined by multivariate logistic analysis.

**Results:**

The ECC rate of children in this study was 57.51%. The mean decayed missing filled teeth (dmft) scores were 3.01. The result of multivariate logistic analysis showed higher ECC prevalence was found in children as age increasing, with lower district economic level, with high frequency of confectionary consumption, having oral medical treatment behavior and bad evaluation of children's oral health by parents.

**Conclusion:**

In general, the prevalence rate of ECC in this study was lower than five years ago, but still higher than those developed countries. And it was associated with age, district economic level, frequency of confectionary consumption, oral medical treatment behavior and evaluation of children's oral health by parents.

## Introduction

In December 2019, an RNA virus belonging to the family Coronaviridae was detected in Wuhan City. It was found to have originated in bats and pangolins and was later transmitted to humans (1). The coronavirus disease can cause toxic pneumonia, fever, cough, myalgia or fatigue, abnormal chest computed tomography, expectoration, headache, hemoptysis, and diarrhea (2–4). This new source of infection is more likely to infect the elderly and cause serious acute respiratory diseases (5, 6). This new viral pneumonia was named “coronavirus disease 2019 (COVID-19)” by the World Health Organization (WHO) (7). According to the WHO's weekly epidemiological update on COVID-19 (January 18, 2022), there have been more than 323 million confirmed cases and over 5.5 million deaths reported worldwide (8), and these numbers are still increasing. Indeed, COVID-19 has had a devastating impact on society. Most countries have implemented various policies to minimize human-to-human transmission, including isolation, blockade, and extensive closures, measures that helped control infection but caused a severe economic contraction (9). A recent study found that the socioeconomic conditions that were compounded by the COVID-19 pandemic were associated with dental pain among the Japanese population. The association related to household income reduction was mediated by psychological distress, postponed dental visits, poor toothbrushing behavior, and between-meals eating behavior (10).

The American Children's Dental Association defines early childhood caries (ECC) as the presence of one or more cavities, missing teeth (due to caries), or fillings on the teeth surfaces of children younger than 71 months (11). ECC is a disease that can lead to the destruction of teeth within a short time, generating conditions so severe that they hinder a child's growth and development. A study has shown that a variety of factors, including social, behavioral, and clinical, may pose risks to the development of ECC (12). In our previous research in 2016, we investigated the related ECC risk factors of children aged 3–5 years in Zhejiang Province. The results showed that older age, region (coastal or mountainous area), higher frequency of bedtime confectionary consumption, and poor care of children's oral health are related to the increased incidence of ECC (13).

Moreover, some studies have assessed the effects of the COVID-19 lockdown on children's oral health. The results of their analyses showed that the frequency of https://www.sciencedirect.com/topics/medicine-and-dentistry/cariogenic-diet/cariogenic-diet consumption increased (14–16), but few studies have investigated ECC prevalence during COVID-19. Therefore, the purpose of this work is to examine the latest oral health status of children, their related behaviors and habits, as well as parents' understanding of oral knowledge and their attitudes toward the oral health of children aged 3 and 5 years in Zhejiang Province. Then, the findings are compared with the research results obtained 5 years ago. Furthermore, the relevant ECC risk factors were analyzed as a basis for improving public health policies for children's dentistry and existing oral health promotion initiatives in response to the COVID-19 pandemic.

## Materials and Methods

### Study Design and Setting

Our cross-sectional study included an oral health examination and an oral health questionnaire. Only children and parents who voluntarily accepted the examination and questionnaire were included in this study. The study was conducted by examining the ECC state of 3- and 5-year-old children in Zhejiang Province between June to November 2021. The parents or caregivers of the surveyed children filled out the structured questionnaire on the day before the examination. All the data were input into the computer for post-analysis. The Oral Health Survey scheme in Zhejiang was approved by the Stomatological Ethics Committee of the Chinese Stomatological Association and the Ethics Committee of Stomatology Hospital Affiliated to Zhejiang University School of Medicine (No. 2021-75).

### Sample Size and Study Participants

The sample size calculation formula is:


$$\begin{array}{l} n=deff\frac{\mu_{a}2p\left(1-p\right)}{\delta^{2}} \end{array}$$

where *n* is the sample size, μ is the level of confidence, p is the dental caries prevalence set at 66.0% (according to the Third National Oral Health Survey), and δ is the margin of error. The design effect *deff* was set at 4.5. The non-response rate was 20%; finally, the minimum required sample size was 1,296 in Zhejiang Province.

The probability-proportional-to-size sampling (PPS) method was used to select children aged 3 and 5 from 11 districts in Zhejiang Province. Specifically, our research selected four kindergartens in each district, for a total of 44 kindergartens. Using the method of quota sampling, the numbers of selected children aged 3 and 5 from every selected kindergarten were 54 and 24, ensuring the same proportion of male and female students as much as possible. A total of 3,537 children and their main caregivers signed an ethical consent and agreed to participate in this study.

### Data Collection (Oral Examination and Survey)

The required instruments for examining the participants' primary teeth included a portable dental examination chair equipped with doctor's seat, artificial light source, community periodontal index (CPI) probes, dental mouth mirror, tweezers, instrument discs, cotton swabs or cotton balls, masks, gloves, medical waste bags, and sharp toolboxes.

An oral examination was performed, and the questionnaire was filled out in strict accordance with the Basic Methods of Oral Health Survey by the WHO (5th edition) (17). The children were placed supine on the portable dental examination chair in their kindergarten. The examiner used an artificial light source to illuminate the mouth and applied cotton balls to wipe the dirt on the tooth surface. Cavities, missing teeth (due to caries), or fillings on the surfaces of teeth were recorded. Each inspection was conducted by three inspectors. The inspectors were trained by a qualified examiner in theoretical knowledge and clinical practice. Then, each inspector enrolled three participants to calibrate the examinations with the qualified examiner. The Kappa value was calculated to be >0.85. Furthermore, 5% of the participating children were randomly re-examined every day to ensure the accuracy of the examination.

On the day before the examination, the parents or caregivers of the tested children filled out the structured questionnaire, and the trained interviewers carefully checked and recorded the responses obtained. The contents included the basic situation of the children, feeding habits, diet-related factors, environmental factors, oral care, parental education, understanding of children's oral health knowledge, and parents' attitudes toward oral health.

### Statistical Analysis

The SPSS 20.0 software (SPSS, Inc., Chicago, IL, USA) was used to analyze the data. To avoid data input errors, the data were entered twice by different researchers. The test level was set at = 0.05. A trend chi-square test was used to analyze whether there were differences in the prevalence of dental caries under disparate variables. We used Mann-Whitney U tests to analyze decayed, missing, and filled teeth (dmft) scores, parents' understanding of children's oral health knowledge scores, and parents' attitudes toward oral health scores. The related variables with ECC were analyzed by multivariate logistic analysis to determine the risk factors, with p < 0.05 in the chi-square test. The variables with *p* > 0.1 were gradually excluded until the remaining variables were all significant.

## Results

A total of 3,537 children and their main caregivers signed the ethical consent and agreed to participate in this study. The response rate was 100%. The numbers of males and females were 1,781 (50.35%) and 1,756 (49.65%), respectively. The numbers of 3- and 5-year-old children were 2,474 (69.95%) and 1,063 (30.05%). Guardians of children filling out the structured questionnaire were mostly mother (72.97%), a few were father (21.74%) and grandparents (5.29%). ECC prevalence (dmft > 0) was 57.51%, which is significantly lower than that in our previous study (70.70%). The mean dmft scores were 3.01 ± 3.89, and the ratio of decayed and filled teeth was 7.04.

The survey results of this study are shown in Table 1. The findings show that the age of the children, residential areas, confectionary (biscuits, cakes, bread, chocolate, sugary gum, etc.), sweet drinks (carbonated drinks, juice, non-freshly squeezed juice, etc.) intake frequency, sweets intake frequency before sleep, age of starting to brush the teeth, brushing frequency, parental help with brushing, medical treatment history of children in stomatology, parents' evaluation of children's oral health, and parental education showed significant associations with the prevalence of ECC (*p* < 0.05).

**Table 1:** Distributions of related variables for ECC.

**Variables**	***n***	**Dental caries**, ***n*** **(%)**		***P*** **-Value**
		**No**	**Yes**	
**Examining time**				
Last study (2016)	2,700	791 (29.30)	1,909 (70.70)	0^***^
This study (2021)	3,537	1,503 (42.49)	2,034 (57.51)	
**Gender**				
Boy	1,781	764 (42.90)	1,017 (57.10)	0.625
Girl	1,756	739 (42.08)	1,017 (57.92)	
**Age, year**				
3	2,474	1,194 (48.26)	1,280 (51.74)	0^***^
5	1,063	309 (29.07)	754 (70.93)	
**Districts**				
Hangzhou	372	203 (54.57)	169 (45.43)	0^***^
Wenzhou	317	173 (54.57)	144 (45.43)	
Yuyao	316	131 (41.46)	185 (58.54)	
Shaoxing	317	114 (35.96)	203 (64.04)	
Jiaxing	312	140 (44.87)	172 (55.13)	
Taizhou	319	148 (46.39)	171 (53.61)	
Jinhua	311	128 (41.16)	183 (58.84)	
Huzhou	318	140 (44.03)	178 (55.97)	
Quzhou	317	89 (28.08)	228 (71.92)	
Lishui	318	104 (32.70)	214 (67.30)	
Zhoushan	320	133 (41.56)	187 (58.44)	
**Guardian**				
Father	769	319 (41.48)	450 (58.52)	0.197
Mother	2,581	1,115 (43.20)	1,466 (56.80)	
Grandparents	187	69 (36.90)	118 (63.10)	
**Feeding types**				
Mainly breastfeeding	2,248	924 (41.10)	1,324 (58.90)	0.078
Mainly formula feeding	576	263 (45.66)	313 (54.34)	
Mixed	713	316 (44.32)	397 (55.68)	
**Confectionary**				
0–3 times/month	634	291 (45.90)	343 (54.10)	0.001^**^
1–6 times/week	1,870	820 (43.85)	1,050 (56.15)	
≥1 time/day	1,033	392 (37.95)	641 (62.05)	
**Sweet drinks**				
0–3 times/month	2,156	993 (46.06)	1,163 (53.94)	0^***^
1–6 times/week	1,123	423 (37.67)	700 (62.33)	
≥1 time/day	258	87 (33.72)	171 (66.28)	
**Sweetened beverage**				
0–3 times/month	1,493	665 (44.54)	828 (55.46)	0.106
1–6 times/week	1,189	490 (41.21)	699 (58.79)	
≥1 time/day	855	348 (40.70)	507 (59.30)	
**Taking sweet before sleep**				
1–4 times/month	1,139	474 (41.62)	665 (58.38)	0.002^**^
2–7 times/week	915	352 (38.47)	563 (61.53)	
Never	1,483	677 (45.65)	806 (54.35)	
**Brushing starting age**				
Before 3	2,623	1,171 (44.64)	1,452 (55.36)	0^***^
3–4 year	646	233 (36.07)	413 (63.93)	
4–5 year	146	41 (28.08)	105 (71.92)	
After 5	44	14 (31.82)	30 (68.18)	
Forgotten	78	44 (56.41)	34 (43.59)	
**Brushing frequency/day**				
≥2	1,374	599 (43.60)	775 (56.40)	0.033^*^
1	2,095	866 (41.34)	1,229 (58.66)	
Don't know	68	38 (55.88)	30 (44.12)	
**Parents' help on brushing**				
≥1 times/day	1,266	585 (46.21)	681 (53.79)	0.001^**^
1–6/week	1,314	554 (42.16)	760 (57.84)	
≤3/month	957	364 (38.04)	593 (61.96)	
**Toothpaste use with Fluoride**				
Yes	1,203	530 (44.06)	673 (55.94)	0.177
No	2,334	973 (41.69)	1,361 (58.31)	
**Dental visit history**				
Yes	1,300	436 (33.54)	864 (66.46)	0^***^
Never	2,237	1,067 (47.70)	1,170 (52.30)	
**Oral health evaluation**				
Excellent	309	200 (64.72)	109 (35.28)	0^***^
Good	1,209	716 (59.22)	493 (40.78)	
Average	1,466	530 (36.15)	936 (63.85)	
Bad	553	57 (10.31)	496 (89.69)	
**Parental education**				
Junior high school and above	811	306 (37.73)	505 (62.27)	0^***^
High school	514	194 (37.74)	320 (62.26)	
Special secondary school/junior college	1,066	445 (41.74)	621 (58.26)	
Bachelor degree or above	1,146	558 (48.69)	588 (51.31)	

* *P* < 0.05.

** *P* < 0.01.

*** *P* < 0.001.

For the personal information of the children, the ECC rate in children aged 3 and 5 years was 51.74 and 70.93%, respectively, which increased with age. In the 11 surveyed districts, the ECC rate in places that had better economies (Hangzhou, 45.43%; Wenzhou, 45.43%) was significantly lower than in other districts. The type or gender of the guardian did not affect the incidence rate of ECC (*P* = 0.197).

In terms of diet-related factors, it was found that a high frequency of confectionary and sweet drink consumption and taking sweets before sleep increased the prevalence of ECC (*P* < 0.05). In addition, no effect of feeding patterns within 6 months after birth (*P* = 0.078) and consumption frequency of sweetened beverages (*P* = 0.106) on ECC rate was found.

In terms of oral healthcare-related variables, the age when children started brushing their teeth, brushing frequency, and parents' help with brushing were associated with ECC prevalence. When tooth brushing started before the age of 3 years, the ECC prevalence was at the lowest (55.36%). Children brushing teeth at least twice (56.40%) had a lower ECC rate than brushing teeth once (58.66%) a day. The ECC rate decreased with an increase in the frequency of parents' help with brushing. In addition, children who had a dental visit history had a higher ECC rate (66.46%). The use of fluoride toothpaste did not affect ECC (*P* = 0.177). The higher the parents' evaluation of their children's oral health, the lower the ECC rate. Moreover, the higher the parents' education level, the lower their children's ECC rate.

Compared with the results obtained in 2016, the prevalence of ECC among children in Zhejiang Province showed a decreasing trend in 2021, with significant changes in eating habits and oral healthcare as shown in Table 2.

**Table 2:** Comparison of eating habits and oral health care between 2016 and 2021.

**Variables**	**2016**	**2021**	***P*** **-Value**
**Dental caries**			
No	791 (29.30)	1,503 (42.49)	0^***^
Yes	1,909 (70.70)	2,034 (57.51)	
**Feeding types**			
Mainly breastfeeding	1,572 (58.22)	2,248 (63.56)	0^***^
Mainly formula feeding	593 (21.96)	576 (16.28)	
Mixed	535 (19.81)	713 (11.68)	
**Confectionary**			
0–3 times/month	541 (20.04)	634 (17.92)	0^***^
1–6 times/week	1,140 (42.22)	1,870 (52.87)	
≥1 time/day	1,019 (37.74)	1,033 (29.21)	
**Sweet drinks**			
0–3 times/month	1,450 (53.70)	2,156 (60.96)	0^***^
1–6 times/week	862 (31.92)	1,123 (31.75)	
≥1 time/day	388 (14.37)	258 (7.29)	
**Sweetened beverage**			
0–3 times/month	815 (30.19)	1,493 (42.21)	0^***^
1–6 times/week	857 (31.74)	1,189 (33.62)	
≥1 time/day	1,028 (38.07)	855 (24.17)	
**Taking sweet before sleep**			
1–4 times/month	1,751 (64.85)	1,139 (32.20)	0^***^
2–7 times/week	372 (13.78)	915 (25.87)	
Never	577 (21.37)	1,483 (41.93)	
**Brushing starting age**			
Before 3	970 (35.93)	2,623 (74.16)	0^***^
3–4 year	220 (8.15)	646 (18.26)	
4–5 year	70 (2.59)	146 (4.13)	
After 5	32 (1.19)	44 (1.24)	
Forgotten	1,408 (52.15)	78 (2.21)	
**Brushing frequency/day**			
≥2	479 (17.74)	1,374 (38.85)	0^***^
1	651 (24.11)	2,095 (59.23)	
Don't know	1,570 (58.15)	68 (1.92)	
**Parents' help on brushing**			
≥1 times/day	292 (10.81)	1,266 (35.79)	0^***^
1–6/week	51 (1.89)	1,314 (37.15)	
≤3/month	2,357 (87.30)	957 (27.06)	
**Toothpaste use with Fluoride**			
Yes	196 (7.26)	1,203 (34.01)	0^***^
No	2,504 (92.74)	2,334 (65.99)	

*** P < 0.001.

For eating habits, in 2021, the proportion of those who administered mainly breastfeeding for 6 months after birth in Zhejiang Province (63.56%) increased compared with that in 2016 (58.22%). The proportion of those who were mainly administered formula feeding decreased. Meanwhile, children who consumed confectionary at least once a day and 0–3 times a month decreased, whereas children who consumed confectionary 1–6 times a week increased. The frequency of children consuming sweet drinks, sweetened beverages, and sweets before sleep decreased.

In terms of oral healthcare, the age at which children started brushing in Zhejiang Province was earlier in 2021 than in 2016. The frequency of brushing teeth per day and parents' help with brushing also increased. The use of fluoride toothpaste showed an increasing trend.

The questionnaire analyzed the parents' understanding of children's oral health knowledge and their attitudes toward oral health through eight and six questions, respectively. The results are shown in Tables 3, 4.

**Table 3:** Response distribution on parents' understanding of oral health knowledge regarding ECC.

**Variables**	***N*** **(%)**	**Dental caries**, ***n*** **(%)**		***P*** **-Value**
		**No**	**Yes**	
**1.Bleeding gums are normal when brushing teeth**				
Right	379 (10.72)	151 (39.84)	228 (60.16)	0.543
Wrong	2,988 (84.48)	1,279 (42.80)	1,709 (57.20)	
Don't know	170 (4.81)	73 (42.94)	97 (57.06)	
**2.Bacteria can cause gum inflammation**				
Right	3,300 (93.30)	1,408 (42.67)	1,892 (57.33)	0.278
Wrong	128 (3.62)	46 (35.94)	82 (64.06)	
Don't know	109 (3.08)	49 (44.95)	60 (55.05)	
**3.Brushing teeth is of no use in preventing gum bleeding**				
Right	432 (12.21)	183 (42.36)	249 (57.64)	0.881
Wrong	2,737 (77.38)	1,168 (42.67)	1,569 (57.33)	
Don't know	368 (10.40)	152 (41.30)	216 (58.70)	
**4.Bacteria can cause dental caries**				
Right	3,032 (85.72)	1,304 (43.01)	1,728 (56.99)	0.056
Wrong	206 (5.82)	71 (34.47)	135 (65.53)	
Don't know	299 (8.45)	128 (42.81)	171 (57.19)	
**5.Taking sugar can lead to dental caries**				
Right	3,130 (88.49)	1,342 (42.88)	1,788 (57.12)	0.363
Wrong	248 (7.01)	95 (38.31)	153 (61.69)	
Don't know	159 (4.50)	66 (41.51)	93 (58.49)	
**6.Dental diseases in primary teeth need no treatment**				
Right	271 (7.66)	118 (43.54)	153 (56.46)	0.002^**^
Wrong	3,052 (86.29)	1,319 (43.22)	1,733 (56.78)	
Don't know	214 (6.05)	66 (30.84)	148 (69.16)	
**7.Pit and fissure sealing can prevent caries**				
Right	2,010 (53.83)	878 (43.68)	1,132 (56.32)	0.241
Wrong	372 (10.52)	149 (40.05)	223 (59.95)	
Don't know	1,155 (32.65)	476 (41.21)	679 (58.79)	
**8.Fluoride is useless in protecting teeth**				
Right	296 (8.37)	126 (42.57)	170 (57.43)	0.029^*^
Wrong	2,256 (63.78)	993 (44.02)	1,263 (55.98)	
Don't know	985 (27.85)	384 (38.98)	601 (61.02)	
1-8.Oral health knowledge score	6.37 ± 1.66	6.45 ± 1.62	6.30 ± 1.69	0.011^*^

* P < 0.05.

** P < 0.01.

**Table 4:** Response distribution on parents' attitude toward oral health regarding ECC.

**Variables**	***n***	**Dental caries**, ***n*** **(%)**		***P*** **-Value**
		**No**	**Yes**	
**1.Oral health is very important to daily life**				
Right	3,511 (99.26)	1,491 (42.47)	2,020 (57.53)	0.285
Wrong	17 (0.48)	6 (35.29)	11 (64.71)	
Don't know	9 (0.25)	6 (66.67)	3 (33.33)	
**2.Regular oral examination is necessary**				
Right	3,419 (96.66)	1,447 (42.32)	1,972 (57.68)	0.368
Wrong	47 (1.33)	20 (42.55)	27 (57.45)	
Don't know	71 (2.01)	36 (50.70)	35 (49.30)	
**3.The quality of teeth is natural and is irrelevant with their own protection**				
Right	223 (6.30)	98 (43.95)	125 (56.08)	0.865
Wrong	3,292 (93.07)	1,395 (42.38)	1,897 (57.62)	
Don't know	22 (0.62)	10 (45.45)	12 (54.55)	
**4.Prevention of dental disease mainly depends on individuals**				
Right	3,421 (96.72)	1,458 (42.62)	1,963 (57.38)	0.643
Wrong	101 (2.86)	40 (39.60)	61 (60.40)	
Don't know	15 (0.42)	5 (33.33)	10 (66.67)	
**5.Protecting the first permanent molar is very important**				
Right	3,442 (96.72)	1,470 (42.71)	1,972 (57.29)	0.256
Wrong	50 (1.41)	16 (32.00)	34 (68.00)	
Don't know	45 (1.27)	17 (37.78)	28 (62.22)	
**6.Bad teeth of mom will affect children's teeth**				
Right	1,377 (38.93)	602	775	0.067
Wrong	2,004 (56.66)	824	1,180	
Don't know	156 (4.41)	77	79	
1-6.Oral health attitude score	5.22 ± 0.68	5.23 ± 0.68	5.21 ± 0.68	0.310

Two questions showed a significant effect on the prevalence of ECC. The accuracy of most questions exceeded 75%, but the question on whether pit and fissure sealing can prevent caries generated only 56.83% while the question on whether the bad teeth of mothers will affect children's teeth garnered 38.93%. To determine whether parents' understanding of children's oral health knowledge and their attitudes toward oral health affected the prevalence of ECC, we assigned each question one point and analyzed the total score of each parent. The results showed that the average understanding of children's oral health knowledge scores of caregivers whose children had caries (6.30 ± 1.69) were lower than those whose children did not have caries (6.45 ± 1.62). Thus, the attitude toward oral health scores showed no effect on the prevalence of ECC.

The variables with *p* < 0.05 were included in the multivariate logistic analysis model. The final analysis results are shown in Table 5. It was found that age, district economic level, frequency of confectionary consumption, oral medical treatment behavior, and evaluation of children's oral health by parents were the related risk factors of ECC.

**Table 5:** Association of relevant variables with ECC, using multiple logistic regression model.

**Variables**	**OR**	**95%CI**		***P*** **-Value**
**Age, year**				
5	Ref			
3	0.48	0.39	0.58	0^***^
**Distracts**				
Hangzhou	Ref			
Wenzhou	1.03	0.70	1.52	0.868
Yuyao	1.82	1.25	2.64	0.002^**^
Shaoxing	2.40	1.64	3.50	0^***^
Jiaxing	1.94	1.35	2.80	0^***^
Taizhou	1.66	1.14	2.44	0.009^**^
Jinhua	2.01	1.37	2.94	0^***^
Huzhou	1.66	1.15	2.40	0.007^**^
Quzhou	3.08	2.09	4.54	0^***^
Lishui	3.21	2.16	4.75	0^***^
Zhoushan	2.04	1.40	2.96	0^***^
**Confectionary**				
≥1 time/day	Ref			
0-3 times/month	0.86	0.67	1.11	0.239
1-6 times/week	0.77	0.63	0.94	0.008^**^
**Dental visit history**				
Never	Ref			
Yes	1.23	1.02	1.49	0.029^*^
**Oral cavity health**				
Bad	Ref			
Excellent	0.06	0.04	0.10	0^***^
Good	0.07	0.05	0.11	0^***^
Average	0.20	0.14	0.30	0^***^

* P < 0.05.

** P < 0.01.

*** P < 0.001.

## Discussion

With the help of the Centers for Disease Control and Prevention and kindergartens, our study, which selected 3 and 5 years old children from kindergartens, had a satisfactory response rate.

Our study was conducted between June to November 2021 during the COVID-19 pandemic. Among the 3,537 children examined in our study, 2,034 had ECC. The prevalence of ECC for children aged 3 and 5 were 51.74 and 70.93%, respectively. The overall prevalence rate (57.51%) was lower than that in 2016 (70.70%). Notably, the ECC rate in Zhejiang Province was higher than in developed countries, such as Japan (3y: 9%; 5y: 39%) (18), the UK (5y: 23%) (19) and USA (2–5y: 21%) (20), and was similar to Thailand (3y: 53%; 5y: 76%) (21) and India (0–6y: 50%) (22). Among the 11 districts in Zhejiang Province, the economically developed districts (Hangzhou and Wenzhou) had a lower prevalence of ECC. Children in developing countries or low-economy districts have a higher prevalence of ECC, which may be attributed to a wide range of social, economic, and political factors, as well as fluoride use and oral health-related habits. In addition, the dentist-to-population ratios and the pediatric dentist-to-children-under-age-5 ratios are small in developing countries, indicating that the insufficient number of dental care professionals is a common problem in these countries, which might affect ECC control (23). The FDI World Dental Federation supports a shift in caries management from restorative treatment to measures that arrest and prevent caries development, such as encouraging the use of preventive dental medicine to reduce the burden of tooth decay. However, thus far, the uptake in daily clinical practice has been slow (24).

Compared with the data from 2016, significant changes have taken place in children's eating habits and oral healthcare in Zhejiang Province in 2021, which is mainly reflected in more breastfeeding and a lower frequency of children consuming confectionary and sweet drinks. Meanwhile, the age at which brushing teeth is initiated is getting earlier, and the frequency of brushing teeth per day, parents' help with brushing, and use of fluoride toothpaste increased. All these changes are conducive to the improvement of children's ECC in Zhejiang Province.

The risk factors of ECC can be biological, behavioral, or socioeconomic contributors to the caries process. The most significant factors contributing to the risk of developing the disease include feeding habits, eating habits, socioeconomic status, oral hygiene practices, levels of *Streptococcus mutans*, the time of the first dental visit, and various dental problems in parents or caregivers (25). According to available evidence, the risk factors of ECC vary among children with different backgrounds and are also affected by the study design, participants, and statistical analysis techniques used in a study (26).

The risk factors of ECC in the present study are age, district economic level, dietary practices, and parents' attention to children's oral health conditions, which are not exactly the same as those found in our previous study. Age was the influencing factor of ECC in both studies, namely, older children have a higher prevalence of ECC. The differences are probably because caries occurrence measures the continuous and cumulative effects of dental caries in the lifetime of a particular dentition (27). Dietary habits play an important role in the development of ECC; when the diet they consume contains high levels of sugar, children are at a high risk of ECC (28) because there are more fermentable carbohydrates in high-sugar foods, which are converted by *S. mutans* into acids that demineralize the enamel and dentin (29).

In the previous study, frequent bedtime sweet consumption significantly increased the risk of caries (13). This study found that the frequency of confectionary consumption affects the prevalence of ECC. There was no significant difference between bottle-fed or breastfed children in both studies, which was similar to the results obtained by other studies (30, 31). However, Shrutha et al. (32) reported that children who were breastfed for a longer duration during nighttime, those falling asleep with a bottle, and those fed with additional sugar in milk have a higher prevalence of ECC. It was also suggested that children who have had a dental visit history have a higher prevalence of ECC. This may be because parents do not care enough about their children's oral health condition, and they will not go to the hospital unless the children's dental caries cause severe pain.

In the questionnaire filled out by the parents, their awareness of children's oral health knowledge and their attitude toward oral health were investigated through eight and six questions, respectively. The findings show that children of parents who know more about oral health are less likely to suffer from ECC. There is a growing body of evidence that reflects the association between parental oral health knowledge and behavior and their child's oral health status (33). Improving primary caregivers' understanding and practices around their children's oral health is central to reducing the existing oral health disparities and enabling sustainable outcomes (34).

This study has some limitations. First, although 11 districts in Zhejiang Province have been included in the study, the community dental education and utilization rate of dental health facilities differ in each district. The four kindergartens randomly chosen by PPS method may not fully represent each district. Finally, either a recall or response bias may have been induced in this cross-sectional study.

## Conclusion

Our study showed that the present prevalence of ECC in children aged 3 and 5 years in Zhejiang Province, China was lower than that obtained 5 years ago. Moreover, it is higher than those in developed countries and similar to developing countries. Age, district economic level, frequency of confectionary consumption, oral medical treatment behavior, and evaluation of children's oral health by parents are the related risk factors of ECC. We suggest that the frequency of confectionary consumption needs to be reduced, and measures should be adopted to increase the dissemination of children's oral health knowledge. The construction of public dental health facilities in various districts should be strengthened for parents and children to access free and high-quality dental services, especially in low-income districts.

## Data availability statement

The raw data supporting the conclusions of this article will be made available by the authors, without undue reservation.

## Ethics statement

The studies involving human participants were reviewed and approved by Stomatological Ethics Committee of the Chinese Stomatological Association and the Ethics Committee of Stomatology Hospital Affiliated to Zhejiang University School of Medicine. Written informed consent to participate in this study was provided by the participants' legal guardian/next of kin.

## Author contributions

JH, HZ, and CH contributed to conception and design of the study. CH, YF, KZ, and LL participated in data collection. CH performed the statistical analysis and wrote the first draft of the manuscript. CH, JH, HZ, YF, KZ, and LL wrote sections of the manuscript. All authors contributed to manuscript revision, read, and approved the submitted version.

## Conflict of Interest

The authors declare that the research was conducted in the absence of any commercial or financial relationships that could be construed as a potential conflict of interest.

## Publisher's Note

All claims expressed in this article are solely those of the authors and do not necessarily represent those of their affiliated organizations, or those of the publisher, the editors and the reviewers. Any product that may be evaluated in this article, or claim that may be made by its manufacturer, is not guaranteed or endorsed by the publisher.
